# Riedel's thyroiditis masquerading as anaplastic thyroid carcinoma: a case report

**DOI:** 10.1186/1752-1947-4-15

**Published:** 2010-01-20

**Authors:** Navneel Shahi, Mohammed F Abdelhamid, Mudit Jindall, Reda W Awad

**Affiliations:** 1 Warwick Hospital, Lakin house, Lakin Road, CV34 5BW, UK; 2 Department of Surgery, Watford General Hospital, WD18 0HB, UK; 3 Department of ENT, University Hospital Coventry and Warwickshire, Second Floor, CV2 2DX, UK

## Abstract

**Introduction:**

Riedel's thyroiditis is a rare thyroid disease characterized by dense fibrous tissues that replace the thyroid gland and invade the adjacent structures that can mimic thyroid malignancy. We discuss the presentation, investigation and management of this very rare condition.

**Case presentation:**

We present a case of a 59-year-old African-Caribbean man who presented with a rapidly growing hard neck mass, a hoarse voice, dysphagia and breathing difficulty that clinically suggested thyroid malignancy. Biopsy, however, revealed dense fibrous tissues suggestive of Riedel's thyroiditis. This was successfully treated with very high dose steroids, with no relapse in the symptoms.

**Conclusion:**

It is important for clinicians to be aware of this diagnosis when managing patients with thyroid disease, because Riedel's thyroiditis can mimic malignancy. In addition, our case demonstrates that this condition should be treated with very high dose steroids to prevent relapse.

## Introduction

Riedel's thyroiditis is a rare chronic inflammatory disease of the thyroid gland characterized by invasion of the thyroid gland and surrounding structures with dense fibrous tissues. It is a very rare condition. At the Mayo clinic, 37 cases were diagnosed in a series of 57,000 thyroidectomies that were performed between 1920 and 1984. The operative incidence was 0.06% and the overall incidence in outpatients was 1.06 per 100,000 [1]. It is most often seen in women. In a review of 178 patients, 83% were reported to be women.

Riedel's thyroiditis is associated with hypothyroidism, hypoparathyroidism, compression of the trachea, larynx, carotid sheath and esophagus. It may also cause invasion of adjacent muscles and mediastinum. Riedel's thyroiditis is also associated with other fibrous inflammatory processes, including retroperitoneal fibrosis, orbital pseudotumour, mediastinal fibrosis, sclerosing cholangitis and fibrosis in other organ systems [2]. We present a rare case of Riedel's thyroiditis that presented a diagnostic dilemma but had a good response to high dose of steroids, with no relapse after a year of treatment.

## Case presentation

**Figure 1:**
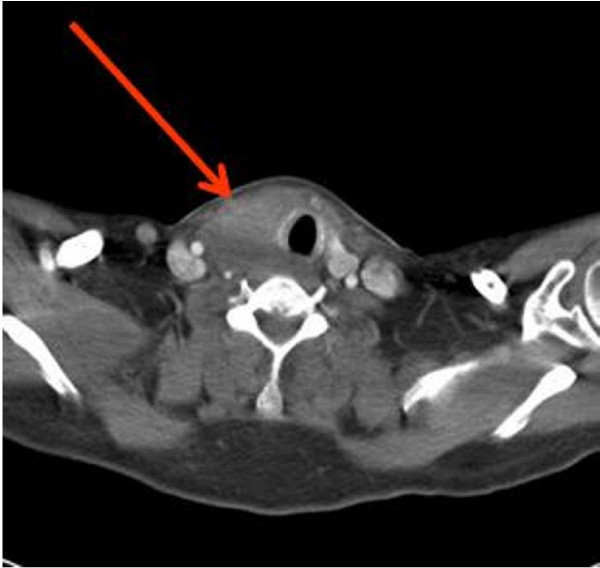
**Computed tomography (CT) scan of the neck and the thoracic outlet**. This shows a large thyroid mass predominantly affecting the right lobe of the thyroid gland. The trachea has been pushed over to the left.

A 59-year-old African-Caribbean man presented with a two-month history of neck pain, hoarse voice, dysphagia and breathing difficulties. He had an anterior neck swelling that increased rapidly in size. Clinical examination revealed an enlarged, hard and fixed goitre that was very tender on palpation. The trachea was deviated to the left side and there was no cervical lymphadenopathy. Fibre-optic laryngeal examination showed reduced mobility of the right vocal cord. Blood test showed a normal blood cell count and calcium and thyroid function tests, negative thyroid peroxidase autoantibodies and an erythrocyte sedimentation rate (ESR) of 95 mm/hr. A computed tomographic (CT) scan of the neck and thoracic outlet showed a large enhancing thyroid mass predominantly affecting the right lobe causing deviation of the trachea to the left with no cervical lymphadenopathy (Figure 1).

**Figure 2:**
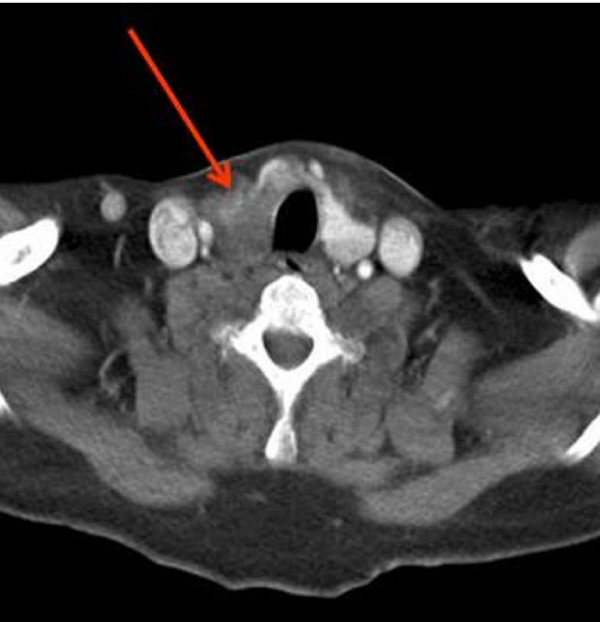
**Computed tomography (CT) scan of the neck and thoracic outlet**. Repeat CT scanning after ten months of steroid therapy confirms reduction in size of the goitre and with return of the trachea to midline position.

During surgery this thyroid mass was found invading all the surrounding structures, including the carotid arteries, internal jugular vein and the right recurrent laryngeal nerve. The trachea was significantly deviated to the left side by this mass and no tissue planes could be identified for dissection. Anaplastic carcinoma of the thyroid was suspected, though unlikely due to the age of the patient. As surgical resection was impossible, a wedge biopsy was taken to establish histological diagnosis. Histopathology showed an admixture of fibrous tissue with focal collagen hyalinization and inflammatory infiltrate rich in lymphocytes and plasma cells. Occasional giant cells were present with atrophic thyroid follicles. At this stage, the diagnosis was confirmed as Riedel's thyroiditis, based on the histology results. The patient was subsequently started on 80 mg/day of prednisolone. After four weeks, there was marked improvement in the voice and swallowing of the patient with reduction in the size of the goitre. Within two weeks of starting steroids, the ESR was noted to have fallen to 10 mm/hr. Prednisolone was gradually tapered over 10 months to a dose of 5 mg on alternate days. Repeat CT scan confirmed reduction in the size of the goitre with minimal shift in the trachea (Figure 2).

## Discussion

The origin of Riedel's thyroiditis remains unknown, although several theories have been proposed. It may be seen as isolated or as part of multifocal fibrosclerosis. In 1922, Ewing stated that Riedel's thyroiditis is a variant of De Quervain's thyroiditis [3]. At one time, it was thought to be an advanced stage of Hashimoto's disease, collagen vascular disorder or an autoimmune disease. Familial occurrence of multiple fibrosclerotic lesions, which include Riedel's thyroiditis, have been reported and have suggested a genetically determined process [4].

The clinical picture of Riedel's thyroiditis is nonspecific and its major clinical significance lies in its ability to mimic invasive thyroid carcinoma [5]. Most patients present with non-painful, non-tender goitre that may appear gradually or suddenly and may produce pressure symptoms such as dyspnea and dysphagia in addition to hoarseness with vocal cord paralysis. However, cervical lymphadenopathy is not present. Our patient was unusual in that his goitre was painful and tender although the rest of the clinical picture was typical.

Laboratory findings in Riedel's thyroiditis are nonspecific. The ESR is frequently elevated. In our patient, ESR was elevated and normalized within two weeks of steroid therapy. Antithyroid antibodies may or may not be positive. In one review, antithyroid antibodies were found in 67% of patients [5]. Antithyroid peroxidase antibodies were negative which is against Hashimoto's thyroiditis. The diagnosis of Riedel's thyroiditis is not possible with fine-needle aspiration because the dense fibrosis precludes adequate aspiration of the gland. Furthermore, the fibrotic reaction can be difficult to distinguish from that surrounding an undifferentiated carcinoma [1].

Surgical intervention is done mainly to establish the diagnosis, exclude malignancy and relieve tracheal compression. Wedge resection of the thyroid isthmus is often adequate to meet both needs. Extensive resection may be difficult due to fibrosis, increasing the risk of injury of vital neck structures such as recurrent laryngeal nerve and carotid artery [6].

There have been no controlled trials on the efficacy of therapy for Riedel's thyroiditis. The condition is extremely rare and the therapy is based on empiric data [7]. There are reports of beneficial effects of steroids in some but not all cases.

In one report, prednisolone (30 mg/day) was used with relapse in the ESR and development of retroperitoneal fibrosis six months after withdrawal of steroids. Relapse after steroid withdrawal has also been reported in Riedel's struma [6]. In our case, we used high dose of prednisolone (80 mg/day) for four weeks, followed by gradual withdrawal. This led to rapid regression of symptoms and reduction in the size of the goitre with no symptoms or signs of relapse or development of other fibrosclerotic lesions.

## Conclusion

In conclusion, we describe a rare case of Riedel's thyroiditis which presented with atypical symptoms of a painful goitre that was tender on palpation. Clinical behavior suggested an anaplastic carcinoma, thus providing a major diagnostic challenge. Although complete surgical resection was not possible, wedge biopsy confirmed the diagnosis. The patient was subsequently started on treatment with very high dose steroid, with good improvement and no recurrence after 12 months.

Finally, it is important for clinicians to be aware of this diagnosis when managing patients with thyroid disease, as Riedel's thyroiditis can mimic malignancy. In addition, our case demonstrates that this condition should be treated with very high dose of steroids to prevent relapse.

## Consent

Written informed consent was obtained from the patient for publication of this case report and accompanying images. A copy of the written consent is available for review by the Editor-in-Chief of the journal.

## Competing interests

The authors declare that they have no competing interests.

## Authors' contributions

NS summarised the patients case notes, did the literature search and was the main contributor in writing the manuscript. MFA contributed to writing the manuscript. MJ edited the manuscript. RA did the final editing of the manuscript. All authors read and approved the final manuscript

## References

[B1] Hay ID Thyroiditis: a clinical update Mayo Clin Proc 1985 60 836 846 3906289 10.1016/s0025-6196(12)64789-2

[B2] Malotte MJ Chonkich GD Zuppan CW Riedel's thyroiditis Arch Otolaryngol Head Neck Surg 1991 117 2 214 217 1991068 10.1001/archotol.1991.01870140102017

[B3] Katsikas D Shorthouse AJ Taylor S Riedel's thyroiditis Br J Surg 1976 63 929 931 10.1002/bjs.1800631210 1009341

[B4] Comings DE Skubi KB Van Eyes J Motulsky AG Familial multifocal fibrosclerosis Ann Intern Med 1967 66 884 892 6025229 10.7326/0003-4819-66-5-884

[B5] Schwaegerle SM Bauer TW Esselstyn CB Riedel's thyroiditis Am J Clin Pathol 1988 90 715 722 3057862 10.1093/ajcp/90.6.715

[B6] Moulik PK Al-Jafari MS Khaleeli AA Steroid responsiveness in a case of Riedel's thyroiditis and retroperitoneal fibrosis Int J Clin Pract 2004 58 3 312 315 10.1111/j.1368-5031.2004.00057.x 15117103

[B7] Dabelic N Jukic T Labar Z Novosel SA Matesa N Kusic Z Riedel's thyroiditis treated with tamoxifen Croat Med J 2003 44 239 241 12698518

